# Supplementary material for: Global economic costs due to vivax malaria and the potential impact of its radical cure: A modelling study
Source: PLoS Med. 2021 Jun 1;18(6):e1003614. doi: 10.1371/journal.pmed.1003614 (PMC8168905; doi:10.1371/journal.pmed.1003614)
Supplement: S2 Table — All costs are in 2017 United States Dollars. (PDF) [file pmed.1003614.s003.pdf]

S2 Table. Regional cost parameters. All costs have been inflated to 2017 United States Dollars.

Cost parameter	WHO region	Base	Low	High	Reference
Malaria rapid diagnostic test	AFRO	$ 3.91	$ 1.96	$ 5.87	[1] Mean +/-50%
	EMRO	$ 1.27	$ 0.64	$ 1.91	[1] Mean +/-50%
	PAHO	$ 1.19	$ 0.60	$ 1.79	[2] Mean +/-50%
	SEARO	$ 2.91	$ 1.46	$ 4.37	[1] Mean +/-50%
	WPRO	$ 1.70	$ 0.85	$ 2.55	[1] Mean +/-50%
Microscopy	AFRO	$ 3.58	$ 1.79	$ 5.37	[1] Mean +/-50%
	EMRO	$ 1.16	$ 0.58	$ 1.74	[1] Mean +/-50%
	PAHO	$ 6.43	$ 3.21	$ 9.64	[3] Mean +/-50%
	SEARO	$ 3.67	$ 1.83	$ 5.50	[1] Mean +/-50%
	WPRO	$ 2.23	$ 1.12	$ 3.35	[1] Mean +/-50%
Fluorescent spot test	WPRO	$ 18.16	$ 9.08	$ 27.24	[1] Mean +/-50%
G6PD rapid diagnostic test	AFRO	$ 3.63	$ 1.81	$ 5.44	[1] Mean +/-50%
	EMRO	$ 3.49	$ 1.74	$ 5.23	[1] Mean +/-50%
	PAHO	$ 4.60	$ 2.30	$ 6.90	[3] Mean +/-50%
	SEARO	$ 3.35	$ 1.68	$ 5.03	Mean of other four regions used for all countries except Indonesia
	Indonesia	$ 15.63	$ 7.82	$ 23.45	[1] Mean +/-50%
	WPRO	$ 1.70	$ 0.85	$ 2.55	[1] Mean +/-50%
Household direct costs	AFRO	$ 2.8	$ 1.5	$ 4.9	[1] Median with 25th – 75th percentiles
	EMRO	$ 3.5	$ 2.1	$ 4.1	[1] Median with 25th – 75th percentiles
	PAHO	$ 14.4	$ 10.0	$ 18.8	[1] Median with 25th – 75th percentiles
	SEARO	$ 3.8	$ 1.8	$ 7.7	[1] Median with 25th – 75th percentiles
	WPRO	$ 2.8	$ 2.6	$ 4.8	[1] Median with 25th – 75th percentiles

References

1. Devine A, Pasaribu AP, Teferi T, Pham HT, Awab GR, Contantia F, et al. Provider and household costs of Plasmodium vivax malaria episodes: a multicountry comparative analysis of primary trial data. Bull World Health Organ. 2019;97(12):828-36. Epub 2019/12/11. doi: 10.2471/blt.18.226688. PubMed PMID: 31819291; PubMed Central PMCID: PMCPMC6883272.

2. de Oliveira MR, Giozza SP, Peixoto HM, Romero GA. Cost-effectiveness of diagnostic for malaria in Extra-Amazon Region, Brazil. Malar J. 2012;11:390. Epub 2012/11/28. doi: 10.1186/1475-2875-11-390. PubMed PMID: 23176717; PubMed Central PMCID: PMCPMC3533805.

3. Peixoto HM, Brito MA, Romero GA, Monteiro WM, de Lacerda MV, de Oliveira MR. Cost-effectiveness analysis of rapid diagnostic tests for G6PD deficiency in patients with Plasmodium vivax malaria in the Brazilian Amazon. Malar J. 2016;15(1):82. Epub 2016/02/13. doi: 10.1186/s12936-016-1140-x. PubMed PMID: 26864333; PubMed Central PMCID: PMCPmc4750282.
