# Supplementary material for: Global economic costs due to vivax malaria and the potential impact of its radical cure: A modelling study
Source: PLoS Med. 2021 Jun 1;18(6):e1003614. doi: 10.1371/journal.pmed.1003614 (PMC8168905; doi:10.1371/journal.pmed.1003614)
Supplement: S1 File — (PDF) [file pmed.1003614.s006.pdf]

S1 File. Equations describing the percent reduction in cases for the radical cure scenarios.

For both scenarios, the first calculation is for the population that would be tested for G6PD deficiency (*pop.tested*) using age bands:

$$pop.tested = 0\text{-}4yr * (1-0.25) + 5\text{-}15yr + [>15yr * (1-preg.lact.exclude)]$$

Note that the age bands (*0-4yr, 5-15yr*, *>15yr*) are for those seeking treatment (incidence multiplied by the percent of cases seeking treatment). A quarter of *0-4yr* are excluded since primaquine should not be prescribed to those under the age of 1. For the *>15yr* age band, women who are pregnant or lactating (*preg.lact.exclude*) are excluded.

It is assumed that primaquine would not be effective in those who do not have G6PD deficiency (*G6PDd*) and are incorrectly prescribed primaquine. Accordingly, the population that could potentially receive an effective dose of primaquine (*PQ.pop.eff*) is calculated. This is the population that are G6PD normal and test normal:

$$PQ.pop.eff = pop.tested * (1 - G6PDd) * specificity$$

where *specificity* is for the G6PD test.

The proportion of patients who are prescribed low-dose primaquine in the baseline analysis (*PQ.prescribed*) and the effectiveness of primaquine without supervision (*unsupervised.eff*) are used to calculate the proportion of patients who are assumed to receive an effective dose of primaquine in the baseline analysis (*baseline.PQ*):

$$baseline.PQ = PQ.prescribed * unsupervised.eff$$

***Supervised radical cure* scenario calculations**

For countries that are not currently prescribing primaquine, the number of cases averted in the *Supervised radical cure* scenario is calculated as:

$$cases.averted = PQ.pop.eff * PQ.efficacy$$

where *PQ.efficacy* is the proportion of recurrent cases prevented when all doses are taken through supervised high-dose primaquine therapy. The number of cases averted for the *Supervised radical cure* scenario for countries that currently prescribe low-dose primaquine is calculated as:

$$cases.averted = PQ.pop.eff * (PQ.efficacy - baseline.PQ)$$

The percent reduction in cases for the *Supervised radical cure* scenario is then calculated by dividing the cases averted by the incidence in the baseline analysis for each country.

***Unsupervised radical cure* scenario calculations**

For countries that don't currently prescribe primaquine in the *Unsupervised radical cure* scenario, the number of cases averted is:

*cases.averted = PQ.pop.eff * unsupervised.eff*

The number of cases averted for the *Unsupervised radical cure* scenario for countries that currently prescribe low-dose primaquine is calculated as:

*cases.averted = PQ.pop.eff * (unsupervised.eff – baseline.PQ)*

The percent reduction in cases for the *Unsupervised radical cure* scenario is then calculated by dividing the cases averted by the incidence in the baseline analysis for each country.
