# Supplementary material for: Global economic costs due to vivax malaria and the potential impact of its radical cure: A modelling study
Source: PLoS Med. 2021 Jun 1;18(6):e1003614. doi: 10.1371/journal.pmed.1003614 (PMC8168905; doi:10.1371/journal.pmed.1003614)
Supplement: S2 File — (PDF) [file pmed.1003614.s007.pdf]

S2 File. Global assumptions, data sources, and distributions for the probabilistic sensitivity analysis.

Table A. Global parameter assumptions and references. All costs are in 2017 United States Dollars.

Parameter	Model value	Low	High	Reference	Notes
Incidence	--	--	--	[1]	See S1 Table.
Treatment-seeking behaviour	--	--	--	[1]	See S1 Table.
Inpatient visit cost (2008)	--	--	--	[2]	See S1 Table. Cost for Somalia was derived from Ethiopia.
Outpatient visit cost (2008)	--	--	--	[2]	See S1 Table. Cost for Somalia was derived from Ethiopia.
Proportion of vivax malaria cases requiring hospitalization	0.02	0.013	0.028	[3]	
Proportion of total *P. vivax* cases confirmed via diagnostic test	--	--	--	[4]	(Number of RDT confirmed cases + Number of microscopy confirmed cases) / Presumed and confirmed cases. For Argentia and Peru, this was assumed to be 100%. See S1 Table.
Proportion of confirmed *P. vivax* cases that are done using an RDT	--	--	--	[4]	Number of RDT confirmed cases / (Number of RDT confirmed cases + Number of microscopy confirmed cases). For Peru, it was assumed that RDT confirmations were also confirmed by microscopy. See S1 Table.
Cost of microscopy	--	--	--	[5, 6]	See S3 Table.
Cost of malaria RDT	--	--	--	[5, 7]	See S3 Table.
Blood stage drug used	--	--	--	[8]	See S1 Table.
Cost of chloroquine	$ 0.28	$ 0.14	$ 0.42	[9]	
Cost of artesunate and amodiaquine	$ 0.92	$ 0.46	$ 1.38	[9]	
Cost of artemether and lumefantrine	$ 1.20	$ 0.60	$ 1.80	[9]	
Cost of dihydroartemisinin-piperaquine	$ 2.87	$ 1.43	$ 4.30	[9]	
Cost of artesunate mefloquine	$1.67	$0.84	$2.51	[10]	

Whether primaquine is recommended in national policy	--	--	--	[4]	See S1 Table.
Proportion of patients who are prescribed primaquine in baseline	0.40	0.10	0.80	Assumption	High bound from [11]. This is applied to those eligible for radical cure (those who are not pregnant/lactating/under 1).
Effectiveness of primaquine without supervision	0.40	0.10	0.70	Assumption	Low bound from [11]
Proportion of recurrent cases prevented by full course of high-dose primaquine	0.88	0.82	0.92	[12]	
Cost of low-dose primaquine	$ 0.38	$ 0.19	$ 0.57	[9]	This is doubled to get the cost of high-dose primaquine.
Proportion of population who are pregnant or lactating	--	--	--	[13]	See S1 Table.
Proportion of under 5s under the age of 1 and excluded from primaquine	0.20	--	--	Assumption	
Proportion with G6PD deficiency	--	--	--	[14]	See S1 Table.
Sensitivity of G6PD test	0.96	0.90	0.99	[15]	Rapid diagnostic test
Specificity of G6PD test	0.95	0.92	0.96	[15]	Rapid diagnostic test
Fluorescent spot test cost	--	--	--	[5]	For Malaysia only. See S3 Table.
G6PD RDT Cost	--	--	--	[5, 6]	See S3 Table.
Household direct costs	--	--	--	[5]	See S3 Table.
Number of days lost for patients	--	--	--	[5]	See S1 Table.
Number of days lost for carers	--	--	--	[5]	See S1 Table.
Number of hours needed for supervision of primaquine	13	--	--		Assuming one hour per visit for 13 days and an 8 hour day.
GDP per capita in 2017	--	--	--	[16]	See S1 Table. Eritrea, Somalia, and Venezuela were derived from [17]. This was multiplied by the number of days lost for illness and needed for primaquine supervision.

GDP Deflators	--	--	--	[18]	Missing years for Djibouti, Eritrea, and Venezuela taken from United States.
Exchange rate for local currency units to United States Dollars	--	--	--	[19]	

G6PD = glucose-6-phosphate dehydrogenase; GDP = gross domestic product; RDT = rapid diagnostic test

Table B. Parameters and distributions used for the probabilistic sensitivity analysis. Low and high values can be found in Table A (above) and in S2 Table.

**Parameter**	**Distribution**	**Notes**
Sensitivity of G6PD test	Beta	
Specificity of G6PD test	Beta	
Proportion of patients who are prescribed primaquine in baseline	Beta	
Effectiveness of primaquine without supervision	Beta	
Proportion of recurrent cases prevented by full course of high-dose primaquine	Beta	
Incidence	Normal	
Treatment-seeking behaviour	Beta	
Mean cost per visit	Gamma	Base value is a combination of inflated inpatient cost (S1 Table), outpatient cost (S1 Table), and proportion of vivax malaria cases requiring hospitalization (Table 1, above). Low and high bounds are +/-50% of base values.
Mean diagnostic test cost	Gamma	Combination of proportion of total *P. vivax* cases confirmed via diagnostic test, proportion of confirmed *P. vivax* cases that are done using an RDT, cost of microscopy, and cost of malaria RDT.
Cost of low-dose primaquine	Gamma	
Cost of blood-stage treatment	Gamma	
Fluorescent spot test cost	Gamma	
G6PD RDT Cost	Gamma	
Household direct costs	Gamma	
Productivity losses for patients	Gamma	Number of days lost is varied before combining with the GDP per capita per day.
Productivity losses for carers	Gamma	Number of days lost is varied before combining with the GDP per capita per day.
Cost of supervision	Gamma	Combination of number of days needed for supervision and GDP per capita per day. The base value used in the PSA was 1.5 days with a low value of 0.25 and a high value of 3.25.

References

1. Battle KE, Lucas TCD, Nguyen M, Howes RE, Nandi AK, Twohig KA, et al. Mapping the global endemicity and clinical burden of *Plasmodium vivax*, 2000-17: a spatial and temporal modelling study. Lancet. 2019;394(10195):332-43. Epub 2019/06/24. doi: 10.1016/s0140-6736(19)31096-7. PubMed PMID: 31229233; PubMed Central PMCID: PMCPMC6675736.

2. World Health Organisation. WHO-CHOICE unit cost estimates for service delivery: WHO; 2011 [cited 2016 02 February]. Available from: http://www.who.int/choice/cost-effectiveness/inputs/health_service/en/.

3. Rahimi BA, Thakkinstian A, White NJ, Sirivichayakul C, Dondorp AM, Chokejindachai W. Severe vivax malaria: a systematic review and meta-analysis of clinical studies since 1900. Malar J. 2014;13:481. Epub 2014/12/10. doi: 10.1186/1475-2875-13-481. PubMed PMID: 25486908; PubMed Central PMCID: PMCPmc4364574.

4. World Health Organization. World malaria report 2018. Geneva: 2018.

5. Devine A, Pasaribu AP, Teferi T, Pham HT, Awab GR, Contantia F, et al. Provider and household costs of *Plasmodium vivax* malaria episodes: a multicountry comparative analysis of primary trial data. Bull World Health Organ. 2019;97(12):828-36. Epub 2019/12/11. doi: 10.2471/blt.18.226688. PubMed PMID: 31819291; PubMed Central PMCID: PMCPMC6883272.

6. Peixoto HM, Brito MA, Romero GA, Monteiro WM, de Lacerda MV, de Oliveira MR. Cost-effectiveness analysis of rapid diagnostic tests for G6PD deficiency in patients with Plasmodium vivax malaria in the Brazilian Amazon. Malar J. 2016;15(1):82. Epub 2016/02/13. doi: 10.1186/s12936-016-1140-x. PubMed PMID: 26864333; PubMed Central PMCID: PMCPmc4750282.

7. de Oliveira MR, Giozza SP, Peixoto HM, Romero GA. Cost-effectiveness of diagnostic for malaria in Extra-Amazon Region, Brazil. Malar J. 2012;11:390. Epub 2012/11/28. doi: 10.1186/1475-2875-11-390. PubMed PMID: 23176717; PubMed Central PMCID: PMCPMC3533805.

8. World Health Organization. World malaria report 2016. Geneva: 2016.

9. MSH (Management Sciences for Health). International Medical Products Price Guide, 2015 Edition. Arlington, VA: 2017.

10. Hill AM, Barber MJ, Gotham D. Estimated costs of production and potential prices for the WHO Essential Medicines List. BMJ Global Health. 2018;3(1):e000571. doi: 10.1136/bmjgh-2017-000571.

11. Douglas NM, Poespoprodjo JR, Patriani D, Malloy MJ, Kenangalem E, Sugiarto P, et al. Unsupervised primaquine for the treatment of *Plasmodium vivax* malaria relapses in southern Papua: A hospital-based cohort study. PLoS Med. 2017;14(8):e1002379. Epub 2017/08/30. doi: 10.1371/journal.pmed.1002379. PubMed PMID: 28850568; PubMed Central PMCID: PMCPMC5574534.

12. Commons RJ, Simpson JA, Watson J, White NJ, Price RN. Estimating the Proportion of *Plasmodium vivax* Recurrences Caused by Relapse: A Systematic Review and Meta-Analysis. Am J Trop Med Hyg. 2020. Epub 2020/06/12. doi: 10.4269/ajtmh.20-0186. PubMed PMID: 32524950.

13. Baird JK, Battle KE, Howes RE. Primaquine ineligibility in anti-relapse therapy of *Plasmodium vivax* malaria: the problem of G6PD deficiency and cytochrome P-450 2D6 polymorphisms. Malar J. 2018;17(1):42. Epub 2018/01/24. doi: 10.1186/s12936-018-2190-z. PubMed PMID: 29357870; PubMed Central PMCID: PMCPMC5778616.

14. Howes RE, Piel FB, Patil AP, Nyangiri OA, Gething PW, Dewi M, et al. G6PD deficiency prevalence and estimates of affected populations in malaria endemic countries: a geostatistical model-based map. PLoS Med. 2012;9(11):e1001339. Epub 2012/11/16. doi: 10.1371/journal.pmed.1001339. PubMed PMID: 23152723; PubMed Central PMCID: PMCPMC3496665.

15. Ley B, Winasti Satyagraha A, Rahmat H, von Fricken ME, Douglas NM, Pfeffer DA, et al. Performance of the Access Bio/CareStart rapid diagnostic test for the detection of glucose-6-phosphate dehydrogenase deficiency: A systematic review and meta-analysis. PLoS Med. 2019;16(12):e1002992. Epub 2019/12/14. doi: 10.1371/journal.pmed.1002992. PubMed PMID: 31834890.

16. The World Bank. GDP per capita (current US$) 2017 [updated 15/10/2020; cited 2020 9 Dec]. Available from: http://http://data.worldbank.org/indicator/NY.GDP.PCAP.CD.

17. UNdata. Per capita GDP at current prices - US dollars: United Nations; 2017 [updated 2020/02/10; cited 2020 9 December]. Available from: http://data.un.org/Data.aspx?q=GDP+per+capita&d=SNAAMA&f=grID:101;currID:USD;pcFlag:true;crID:232,408,410,807,862;yr:2017&c=2,3,5,6&s=_crEngNameOrderBy:asc,yr:desc&v=1.

18. The World Bank. Inflation, GDP deflator (annual %) 2009-2017 [updated 15/10/2020; cited 2020 9 December]. Available from: https://data.worldbank.org/indicator/NY.GDP.DEFL.KD.ZG.

19. The World Bank. Official exchange rate (LCU per US$, period average) 2017 [updated 15/10/2020; cited 2020 9 Dec]. Available from: https://data.worldbank.org/indicator/PA.NUS.FCRF.
