# Supplementary material for: Global economic costs due to vivax malaria and the potential impact of its radical cure: A modelling study
Source: PLoS Med. 2021 Jun 1;18(6):e1003614. doi: 10.1371/journal.pmed.1003614 (PMC8168905; doi:10.1371/journal.pmed.1003614)
Supplement: Attachment — Submitted filename: Response to review (Global cost).docx [file pmed.1003614.s008.docx]

**Requests from the editors:**

**1.	Please address the reviewers’ and academic editor’s comments below.**

We have addressed each comment specifically below.

**2.	Please revise your title according to PLOS Medicine's style. Your title must be nondeclarative and not a question. It should begin with main concept if possible. "Effect of" should be used only if causality can be inferred, i.e., for an RCT. Please place the study design ("A randomized controlled trial," "A retrospective study," "A modelling study," etc.) in the subtitle (ie, after a colon).**

The title has been revised to:

“*Global economic costs due to vivax malaria and the potential impact of its radical cure: A modelling study*”

**3.	Abstract:**

**a.	Around lines 49-50: Please include date ranges for your data.**

This has been rephrased to provide clarity:

*“Estimates of the healthcare provider and household costs due to vivax malaria were collated and combined with national case estimates for 44 endemic countries in 2017.”*

**b.	Please perhaps include estimates of patients affected by vivax malaria, and a break-down by world regions.**

We have added this to the abstract:

*“In 2017 an estimated 14 million cases of Plasmodium vivax malaria were reported from Asia, Central and South America, and the Horn of Africa.”*

**c.	Please rename the “Interpretation” section to “Conclusions”. Please begin this section with “In our modelling study, we found that…”**

This has been changed.

**4.	Author Summary: Please briefly explain “vivax malaria” to a lay reader.**

We have expanded on the first bullet point as follows:

*“Vivax malaria is a mosquito-borne febrile illness common in Asia, Latin America, and the Horn of Africa. Although a cause of significant morbidity, its global cost burden has not previously been estimated using country-level data.”*

**5.	Please remove spaces from within citation callouts, eg: “…and indirect attributable mortality [4,5] and ongoing…”**

The spaces within citation callouts have been removed throughout the text.

**6.	Methods: Around lines 155-169: Please include date ranges for your data.**

This has been revised to clarify that it’s for the year 2017:

*“The Malaria Atlas Project estimated that the incidence of vivax malaria in 2017 was 14.2 million cases across the 44 endemic countries included in this analysis (S1 Table) [2]. These estimates refer to symptomatic vivax malaria and were used as the time horizon for the costs from the healthcare provider and societal perspectives. Estimates utilise treatment-seeking rates at public facilities to adjust for cases that would not be included in national reporting systems due to individuals attending private healthcare providers, or never seeking treatment. National estimates of treatment-seeking behaviour were derived from household survey data [21] that were categorized according to whether patients sought treatment with any provider (including public or private healthcare providers, pharmacies, or shops) or did not seek treatment outside of their own home. Treatment-seeking values were modelled for countries and years without household data using socioeconomic indicator variables and a Gaussian process regression [2]. Case values for 2017 were also adjusted for reporting completeness using subnational values publicly available from country programs, or national values as reported in the World Malaria Report [22]. Age-specific incidence rates were derived from a model originally calibrated for P. falciparum but adapted for P. vivax [23,24]. Case estimates for 2017 were available for all endemic countries, except for the majority of sub-Saharan Africa due to a paucity of case data.”*

**7.	Line 388: Please clarify here: “To our knowledge, this paper collates for the first time…”**

This has been added to the discussion.

**8.	Please remove this text from reference 44: “coordinating a workshop in Port Moresby to provide advice on mathematical modelling to the Papua New Guinea National Malaria Control Programme. M.T.W., P.W. and A.G. declare that they have no other competing interests. All remaining authors declare no competing interests.”**

This has been removed from the reference.

**9.	Please ensure that the study is reported according to the CHEERS guidelines, and include the completed checklist as Supporting Information. When completing the checklist, please use section and paragraph numbers, rather than page numbers. Please mark itens not applicable as “n/a”. Please add the following statement, or similar, to the Methods: "This study is reported as per the Consolidated Health Economic Evaluation Reporting Standards (CHEERS) guideline (S1 Checklist)."**

The statement has been added at line 173 with the checklist included as S2 Checklist. As clarified in the text, this is not a comprehensive CEA, so we have completed this checklist as best as we can.

**Academic editor feedback:**

**1. I agree with the first reviewer that GDP/capita/day probably underestimates the value of clinical providers' time. More important, though, is that it may also incorrectly value households' time, which makes up a fair share of the cost savings identified. From a colleague's paper about the cost of childhood malaria in Kenya: "Per capita income underestimates adult income because children are included in the calculation. However, per capita income overestimates average income because the distribution of income in Kenya is very skewed: 20% of the country's total population is estimated to earn 50% of national income." (Source: PMID 17196105). I would ask the authors to use a more thoughtful estimate of the value of time and to allow it to vary between providers and patients. The paper I cite here has some suggestions for how to do that. Even just providing examples of how this would affect results for a few of the highest burden countries would help.**

In many settings, this work would be done by unpaid community health workers, and so we valued this time in the same way that we did the patients. We have revised the methods to better describe this justification. Line 234:

*“Since most community healthcare workers are unpaid, their time was valuated using the GDP per capita per day [35,36].”*

Regarding the value of children’s time, we did not assign a productivity loss to those under 5 years, which should mitigate some of the concerns around whether this was over-estimated. To explore the effect of this further we have included a sensitivity analysis in which only productivity losses to adults were included. We agree with the reviewer and editor that this is a contentious area and conducted extensive discussions regarding valuing productivity losses for unpaid workers amongst ourselves and with colleagues not involved in this study, and the consensus was that using 1 GDP per capita per day for unpaid labour is the best (albeit imperfect) option currently available for low- and middle-income settings. While the method presented in Larson 2006 is an interesting option, our concern is that applying different income levels to providers and patients (or to different demographics of patients) could inadvertently exacerbate inequities. Productivity losses in someone in unpaid work may not have a high financial cost, however, it can have profound social consequences when that labour is required for critical functions such as caring for the family.

**2. The issue of “cost to whom,” while mentioned, is not addressed sufficiently in the discussion. Treating vivax malaria substantially increases costs to providers. All the savings are to households, and a large share of these savings are not cash in hand (i.e. they are indirect benefits of not losing productive time). As the authors note, this makes the intervention look like a bad deal to governments. It also masks the value to households, which might appreciate the saved time but are unlikely to count it as an economic benefit unless there is an immediate income effect. A stronger discussion of how to frame interventions that impose an immediate financial cost on the provider to generate future benefits to patients would be welcome.**

Most health interventions have immediate financial costs for the healthcare providers whilst providing benefits for the patients (it is relatively uncommon for new interventions to save costs for the healthcare providers and provide incremental health benefits for patients). The key question is whether those incremental provider costs are considered a good investment as compared with the health gains to patients. Most cost-effectiveness analyses in LMIC contexts will use the cost per DALY averted for these assessments. As our primary objective in this work was to carry out a global costing of vivax malaria, rather than a detailed cost-effectiveness analysis of specific radical cure interventions, we felt that focusing on the total costs in the different scenarios is appropriate. To clarify who is incurring the costs, we report provider costs, direct household costs, and indirect household costs separately throughout the manuscript. The second paragraph of the discussion now reads:

*“Our analysis estimates the current global societal costs of vivax malaria and the prospects for reducing these costs if radical cure strategies were to be widely implemented; it is not, however, a comprehensive cost-effectiveness analysis for the introduction of radical cure strategies and programmes, which would require consideration of other factors and further contextual and country-specific adaptations. Changing antimalarial policy and practice, for instance, would incur further investment in implementation activities, including training and strengthening supply lines, and these costs can be substantial [41,42], and should be included in the country-level cost-effectiveness analyses. The increased healthcare provider costs that we describe alongside the additional resources needed to implement the policy changes might present a major challenge for sustained financing and a disincentive to changing national policy. To put this in context, however, the additional US$39 million provider expenses required for global G6PD screening and treatment represents only 1% of the US$3.1 billion spent on malaria control activities in 2017; an investment that could potentially halve the global burden of P. vivax. Since a large proportion of vivax malaria episodes are attributable to relapses [43,44], investment in safe and effective radical cure will be crucial to achieving the timely elimination of vivax malaria.”*

**---**

**Comments from the reviewers:**

**Reviewer #1:**

**Malaria eradication remains a global challenge despite numerous programs and initiatives to accomplish it, and this paper contributes positively by suggesting a fresh angle of attack to this problem. I also agree with the authors that P. vivax has probably received too little attention, and see this manuscript as a potentially important contribution. I also think the main conclusion is plausible that provision of a safe and radical cure is possible, and that it indeed will require and increased investment for national malaria control programs. However, I have some comments to the methods which in consequence means that I think this paper underestimates both the required investments and perhaps overestimate potential benefits. I will present the comments in an order which I perceive as decreasing importance:**

**1. My most substantial comment is about the costing of health providers, which are important since they represent the actual investments national health services need to make. The authors have chosen a societal perspective for cost estimation, which is commendable. They include direct and indirect household cost, as well as direct provider costs. The challenge, as I see it, is that the latter appears to be based on a rather narrow identification of provider cost items and therefore probably is underestimated. Provider costs are sometimes limited to facility level, which can be meaningful when comparing health interventions at facility level. The scope of this paper is however to discuss national scale-up of (i) screening and (ii) subsequent treatment. This will require national level efforts to build competence, set up routines for the different procedures and provide and monitor treatment. For example, training is required of the different cadres of laboratory and other health workers to facilitate these interventions. In order to provide repeated supervision of treatments, a system with village health workers need to be operational, sensitised and trained, and their availability probably varies substantially between the included countries. In sum, it appears that none of these systemic costs of scaling up these interventions have been evaluated. This seems apparent in Table 2, where the total provider costs for low-incidence countries such as Belize, Bhutan, China, El Salvador, Iran and Timor-Leste accumulate to between 50 and 200 USD. I recommend that the range of provider costs is re-evaluated in order to reflect more realistic measures for investments of the public health systems.**

We agree that additional provider costs for policy change, training, and delivering novel strategies for radical cure could be substantial and have not been included in our analysis. We have revised the text to highlight this major limitation in lines 424-439 as per Academic Editor’s comment #2 and also in the limitations (line 485):

*“The cost of scale up required to achieve provider compliance with G6PD screening and radical cure are also not included in this time frame, underestimating the cost of implementation.”*

**2. Also pertaining to provider cost is the assumption (line 229) that health workers time is valued using GDP per capita per day. While I think this is a reasonable assumption for national level valuation of productive time, I think this is an underestimate when applied to health workers. Many of the included countries have large informal sectors, such as subsistence farming and petty trade, with generally very low levels of income per capita that bias the national GDP estimates per capita downward. Health workers on the other hand belong to the formal sector, where the income levels are generally substantially higher.**

We recognize that we didn’t make it clear that these workers would be part of the informal sector and have clarified this in the methods (Line 234):

*“Since most community healthcare workers are unpaid, their time was valuated using the GDP per capita per day [35,36].”*

**3. Figure 1 is a flow chart illustrating the structure of procedures and costs. Early in the model, eligibility is considered if "patient is not pregnant, lactating or under the age of 1 year". This state may lead to G6PD testing and subsequent treatment. It is however confusing that exactly the same eligibility criteria are repeated further down in the procedures. Why this replication?**

We were attempting to show how the cases averted were calculated, but the figure reflected this process poorly. We have revised the figure to show the calculations for the radical cure scenarios.

**4. A different but related confusion is in lines 248-250, where it is explained that the cost of testing was applied to the patient population eligible to receive primaquine. I would expect that the cost of testing applied to all that were tested, not just those with an eligible test result.**

All patients who were over the age of 6 months and not pregnant or lactating received a G6PD test and the cost of this test is included in the cost estimates. We have clarified that the eligible patient population here relates to demographic exclusions, not G6PD deficiency:

*“The cost of a G6PD RDT, including an additional blood draw, was applied to the patient population eligible to receive primaquine (i.e. not pregnant, lactating, or under the age of 6 months).”*

**5. In line 292, the administration of an anti-relapse dose is explained. This would become clearer if this outcome and procedure was described in the introduction.**

We have added further details to the introduction (line 124):

*“Adherence to such a prolonged course of treatment for an acute febrile illness is poor, resulting in a high proportion of patients prescribed unsupervised primaquine in routine clinical practice receiving a dose that is ineffective for radical cure [11,12].”*

**6. The paper considers human transmission of P. vivax, but does not mention bovine transmission. How could the presence of bovine transmission influence on the results and conclusion?**

We acknowledge in our discussion that our study does not account for the impact of primaquine on transmission; however, as stated in the discussion, this means that our cost estimates are conservative. With regard to bovine transmission, the reviewer may be referring to *Trypanosoma vivax* which is endemic among bovines in some areas, while *Plasmodium vivax* only infects primates, principally humans.

**7. Line 173 states "RDT or microscopy", while line 175 states "RDT and microscopy". Where both or only one of the procedures performed? This has relevance for costing.**

At most, one test was performed per person. We now include further details on these calculations:

*“For patients seeking treatment at healthcare providers, the proportion of malaria cases diagnosed by either rapid diagnostic test (RDT) or microscopy as well as the drugs prescribed in 2017 were derived for each country from the World Malaria Report; S1 Table [22]. After applying the percent of cases confirmed by diagnostic test to the population seeking treatment, the percent of confirmed cases diagnosed by RDT was used to calculate the RDT costs whilst the cost of microscopy was applied to the remaining individuals.”*

**8. Line 189: Please expand acronym FST on its first (and only?) appearance.**

We have removed this acronym throughout the text.

**9. The paper use US$ exchange rates from 2016. This is fine, but it would have been more eloquent (and more update) to use 2017 rates, since this is also the base year for incidence estimates.**

Agreed. We have updated to 2017 US$ throughout to align with the incidence.

**Producing such a paper based on macro-level evidence is challenging, and will always entail many trade-offs between availability and quality of data. I believe this is something we must accept and that research such as this has merit and is of potential importance for policy formulation. The value of such research, in my opinion, lies primarily in its ability to provide a birds perspective of important public health issues rather than representing exact information at local levels. With some methodological improvements, I think this paper is publishable.**

Thank you.

**Reviewer #2:**

**This interesting manuscript describes the annual global cost of Plasmodium vivax malaria and estimates the cost savings associated with G6PD screening and radical cure assuming perfect and 30% adherence to treatment. Overall, it is an important topic that represents a contribution to the evidence based, but the analysis will be strengthened by addressing the comments noted below.**

**Adherence scenarios:**

**30% adherence is considerably lower than the adherence documented for malaria treatment in most settings. As such, stronger justification should be provided for the selection of that level or a more reasonable alternative scenario should be described.**

Estimates of adherence are notoriously difficult to gather, due to the Hawthorne effect and change in behaviour when patients are observed. Most values of adherence derived from clinical trial settings are overestimates. The Bruxvoort systematic review that we now cite only has one study on adherence to 14-day primaquine regimen in Thailand that was 25% while the 5-8 day primaquine regimens ranged from 62-86%. We use 40% effectiveness for unsupervised primaquine. This is a combination of patient adherence and the efficacy of primaquine in those who are adherent. It’s also important to note that while we know that adherence to the shorter blood-stage treatments and the 7-day primaquine regimen is higher, this is for a 14-day primaquine regimen. We have added the following (line 235):

*“In the second scenario, Unsupervised radical cure, primaquine radical cure in G6PD normal patients was assumed to have only 40% effectiveness due to lower patient adherence. Limited information is available on adherence to and effectiveness of a 14-day primaquine regimen [11,38].”*

**Also- the abstract states 30% adherence, but the methods section (line 231 and 241) describes 40% adherence. Please align and clarify what was used. As noted above, a 40% figure seems more appropriate.**

The abstract has been corrected to state 40% effectiveness (due to a lower rate of adherence) for unsupervised primaquine.

**Cost time horizon:**

**The analysis considers the one year cost savings associated with the intervention. Given that the goal of mass screening and administration of radical cure throughout endemic countries, it is reasonable to assume that subsequent years would have further reductions in vivax transmission and, in turn, further savings. This should be better described within the limitations section.**

This has been added to the limitations in the discussion (line 483):

*“The scenario analyses only included costs over a one year time horizon; accordingly, relapses prevented beyond the time frame are not captured, underestimating the cost savings.”*

**Additionally, interventions to improve care-seeking will also improve the impact of the proposed intervention.**

We do not propose interventions to improve care-seeking, though with better care offered in the public sector through radical cure, it is likely that treatment-seeking behaviour would shift to the public sector.

**Supplementary Table 1- Costs for primaquine in each country should be added alongside the cost of RDT. Please confirm that these costs are currently included in the scenarios as it would be an important additional cost of introduction of radical cure.**

The cost of primaquine is a global assumption as we don’t have country-level cost data. This can be found in Table 1 of S3 file, but we have now added it to S1 Table for the countries that have primaquine as a policy. The cost of primaquine is included for countries with a policy of primaquine use in the baseline estimates and for all countries in both radical cure scenarios. The way that the cost of primaquine is applied in the base case is found in lines 185-195:

*“In the 42 countries in which primaquine was recommended in national guidelines [22], this was assumed to be in line with the WHO Antimalarial Treatment Guidelines, in which treatment is only recommended to non-pregnant and non-lactating females and children over the age of one year [10]. Accordingly, the estimated proportion of patients who were pregnant or lactating were excluded from those over the age of 15 [25] and 20% of cases under the age of five were excluded from primaquine eligibility. From experience in the field, provider compliance to national treatment guidelines for eligible patients was assumed to be 40%, and this was applied to the eligible population to determine the number and associated cost of primaquine prescriptions. In 2017, only Malaysia routinely assessed G6PD status prior to primaquine administration; accordingly, the cost of a fluorescent spot test was applied to all patients eligible for primaquine and the cost of primaquine was added for the proportion who were G6PD normal.”*

We have clarified that the cost of primaquine is included in both radical cure scenarios (line 228):

*“In both scenarios, the cost of a high total dose of primaquine (7mg/kg) was used because it has proven high efficacy across multiple and diverse locations; however, this potentially overestimates the cost of primaquine in areas where a lower total dose may have sufficed [37].”*

**Minor comments:**

**Line 220- I am assuming that the analysis assumes only screening patients who are symptomatic (or positive?) for vivax malaria. This should be explicitly stated.**

The reviewer is correct. Battle et al (2019) state that their estimates are for symptomatic/clinical vivax malaria. We have stated that explicitly here as well by adding to the sentence starting at line 158 in the Methods:

*“These estimates refer to symptomatic vivax malaria and were used as the time horizon for the costs from the healthcare provider and societal perspectives.”*

**Line 228- what was the rationale / evidence for using ¼ of a healthcare worker's time per a case? This seems quite high.**

This was chosen to be conservative when estimating the costs of the radical cure scenario, but we agree that this is likely too high and have lowered this to one hour per visit:

*“The cost of treatment supervision was estimated to be 1.6 healthcare worker days (one hour per day for 13 days assuming a work day of 8 hours). Since most community healthcare workers are unpaid, their time was valuated using the GDP per capita per day [35,36].”*

As mentioned in the discussion, this cost could be further lowered through other follow up methods, such as phone calls and text messages.

**Figure 2- the blues used to denote the most expensive and second most expensive categories are very similar. I suggest changing to make it easier to differentiate India from the other countries.**

We have switched color schemes for Figure 2 and hope that this makes the categories easier to distinguish.

**Table 2- it would be helpful to include an additional column with average cost per a case for each country to provide more evidence about the varied costs by country referenced in line 307.**

Line 307 is referring to the total cost burden, not the individual burden. The provider cost per case is found in S4 Table. Direct household cost per case, patient productivity costs and carer productivity costs are in S1 Table. Since the rest of Table 2 is country-level costs, we think the inclusion of the average cost per case might confuse the reading of the table, therefore instead we now reference in the legend that the provider cost per case is listed in S4 Table.

**Line 466 Provision of access to the online application is very useful and an important contribution.**

Thank you! We agree that with the many assumptions underlying this work (and other similar studies) these applications are important to allow readers to explore the impact of varying the assumptions and parameter estimates.

**Reviewer #3:**

**This study is concerned with an interesting subject, the cost of vivax malaria. While the general question is important and the methodology is sound, there is a number of issues which require clarification and amendment. Specific comments are given below.**

**A major choice in this exercise relates to the selection of the cure method. The authors focus exclusively on primaquine and appear to downgrade the option of treating patients with tafenoquine, which is only briefly mentioned throughout the paper, including a note in the discussion. This is a central/strategic option since using tafenoquine instead would alleviate the need for adherence in practice and the corresponding assumptions in this modelling study. Hence, if the authors will not add the options of treating patients with tafenoquine, a more extensive discussion is in order, with the relative pros and cons of the two treatments, including the option of meticulous (e.g. repeated) testing for G6PD deficiency combined with the use of tafenoquine.**

We agree that a single dose regimen such as tafenoquine would offer huge promise for widespread radical cure. The challenges however for its use are considerable therefore at this point we focused our analysis on the strategies we consider currently available. We have added further details on this decision in the discussion (lines 436-445):

*“The introduction of single-dose tafenoquine provides another alternative that avoids the challenges of compromised adherence; however, this will require more stringent and costly diagnosis of G6PD deficiency with a quantitative test to exclude treatment of individuals with intermediate or severe deficiency (<70% enzyme activity). Routine quantitative G6PD testing requires hand-held devices to be placed at healthcare facilities, adding significant provider costs. Since these costs will vary considerably with patient throughputs and which levels of healthcare facilities the devices are utilized at, we were unable to include them in our analysis. Until tafenoquine and quantitative testing become widely available, primaquine will continue to be the standard of care; and thus complementary interventions to improve adherence will be critical to malaria elimination efforts.”*

**The authors describe their approach reasonably well. However, from a cost-effectiveness standpoint the results are somewhat mixed in that the cost is split, preventing from the reporting of traditional measures like per country ICERs and Incremental Net benefit. Can the authors possibly report such measures, perhaps accounting for the type of country-specific healthcare systems?**

We did not set out to do a comprehensive cost-effectiveness analysis, as our focus was on capturing the global costs of vivax malaria in the current status where radical cure is not widely implemented, and compare these with the costs of hypothetical scenarios where radical cure strategies are widely used (clarified further in the text). As stated in other reviewer comments, switching over to these strategies would incur considerable expenses. This will need to be considered alongside further country-specific specific adaptations required for a comprehensive cost-effectiveness analysis. Since this was not our objective, we do not report ICERs or other standard measures used in CEAs

**The authors chose not to use an epidemic model for estimating the benefit of the proposed approach, therefore underestimating the benefit of indirect protection. I understand that using a full epidemic model may be out of the scope of the present paper, but wonder if the authors could use a multiplier for incorporating the indirect effect as a sensitivity analysis, thus giving a rough order of the complete benefit of the proposed measures, instead of simply calling it a conservative approach.**

We agree! A more in depth analysis including the impact of radical cure on transmission was beyond the scope of the current analysis. Simply adding a multiplier for transmission benefits would add further uncertainty to the estimates, since the impact on transmission would be dependent on incidence, which varies geographically, both between and within countries. However, we highlight this limitation in the discussion (lines 446-455) and emphasise that if anything this would imply the results of our analysis are conservative.

**It would be beneficial to add a table with the critical inputs, including the assumed sensitivity and specificity for each test, giving a sense of their importance in the output of the model.**

All of our model parameters are listed in S1 Table, S3 Table, and S4 File. Table 1 summarizes the key epidemiological inputs by region.

**Please add a short discussion point regarding the completeness of capturing all the vivax cases and how the treatment-seeking reports may vary by country.**

This is an important point and we have added to the Discussion starting at line 463 following mention of uncertainty introduced by adjusting for reporting completeness:

*“Case counts are scaled up based on the estimated treatment seeking rates in each country. The rate of seeking care and percentage of this which occurs through facilities that are integrated into the health management information systems varies widely between vivax endemic countries [49].”*

**The data in table 1 are slightly puzzling, having the eastern Mediterranean region (EMRO) as the one with the second highest burden. From inspecting the countries of table 2 (and table 4) it is unclear which of those are located in EMRO, is that a typo?**

S1 Table presents the WHO region for each country. The high number of cases in EMRO is due to Pakistan.
