# Supplementary material for: Global economic costs due to vivax malaria and the potential impact of its radical cure: A modelling study
Source: PLoS Med. 2021 Jun 1;18(6):e1003614. doi: 10.1371/journal.pmed.1003614 (PMC8168905; doi:10.1371/journal.pmed.1003614)
Supplement: Attachment — Submitted filename: R2 Response - global cost v2.docx [file pmed.1003614.s009.docx]

**Requests from Editors:**

**- Please use the "Vancouver" style to reformat reference #10 and include a link to the online source. See our website for other reference guidelines** [**https://aus01.safelinks.protection.outlook.com/?url=https%3A%2F%2Fjournals.plos.org%2Fplosmedicine%2Fs%2Fsubmission-guidelines%23loc-references&amp;data=04%7C01%7Cangela.devine%40menzies.edu.au%7Ce951d6fc754c4d61a65008d8ef9ee5ad%7C9f2487678e1a42f3836fc092ab95ff70%7C0%7C0%7C637522813040644607%7CUnknown%7CTWFpbGZsb3d8eyJWIjoiMC4wLjAwMDAiLCJQIjoiV2luMzIiLCJBTiI6Ik1haWwiLCJXVCI6Mn0%3D%7C1000&amp;sdata=HtVFLvPx1kc0Ip%2B%2FYhbrq4tC4xIUOsWWKyK2f3diwUo%3D&amp;reserved=0**](https://aus01.safelinks.protection.outlook.com/?url=https%3A%2F%2Fjournals.plos.org%2Fplosmedicine%2Fs%2Fsubmission-guidelines%23loc-references&amp;data=04%7C01%7Cangela.devine%40menzies.edu.au%7Ce951d6fc754c4d61a65008d8ef9ee5ad%7C9f2487678e1a42f3836fc092ab95ff70%7C0%7C0%7C637522813040644607%7CUnknown%7CTWFpbGZsb3d8eyJWIjoiMC4wLjAwMDAiLCJQIjoiV2luMzIiLCJBTiI6Ik1haWwiLCJXVCI6Mn0%3D%7C1000&amp;sdata=HtVFLvPx1kc0Ip%2B%2FYhbrq4tC4xIUOsWWKyK2f3diwUo%3D&amp;reserved=0)

The references have been reformatted as requested and a link is now included.

**- Please update reference #3**

This has been updated.

**Comments from Reviewers:**

**Reviewer #2:**

**No additional comments**

**Reviewer #4:**

**This manuscript provides estimates of the global costs associated with treating P. vivax malaria, and the potential benefit of expanding access to radical cure with primaquine. This manuscript had already been on a journey by the time it landed on my desk, having been read by three other reviewers, with the authors consequently undertaking substantial revisions. Having read the manuscript, the reviewers' comments, and the authors' response, I have found that the majority of the concerns that I would have had have already been addressed by the other reviewers - I thought they did a very thorough job. Nonetheless, I do have some minor comments.**

**On a more general note, an analysis such as this is undeniably hard, and requires an enormous number of assumptions. Some of these assumptions may be inappropriate or downright wrong - such is the case with all model-based analyses. However, I do find the authors major assumptions to be reasonable and in line with current best practices for this sort of global health analysis. Whether current practices in global health analytics are up to scratch is a different debate. I would probably have done some things differently (e.g. demography, treatment effectiveness, adherence, etc.) but I don't believe any of these would substantially alter the work's findings. At the previous reviewers' request, the authors have included sensitivity analyses, and I find these to be satisfactory. It is tempting to ask for another 100 sensitivity analyses, but I don't think this would bring any additional value.**

**In summary, I think this is a solid analysis whose qualities, and flaws, are in line with current best practices in large global health analytics.**

**Minor comments**

***	The online model is a very nice tool.**

Thank you.

***	Please take care when referring to confidence versus credible intervals. There is a bit of inconsistency.**

We have carefully reviewed the manuscript again but cannot identify any issues with these. The only mention of confidence intervals in the text is at line 281 where we describe that the ranges that were sampled from in our PSA were taken from confidence intervals whenever possible.

***	I think there's something wrong in Table 1. There may have been a mix up between the first and second iterations of the manuscript (bottom two rows). Please take a very careful look at this.**

Thank you for bringing this to our attention. There was an issue with the transcription of our data that has now been corrected.

***	I found Table 3 quite unintuitive and it took me a while to work out what was going on. I preferred the format in the first iteration which broke the analyses into several columns.**

Agreed. We have amended to use the same table formatting with several columns from the first iteration.

***	The estimates in Table 4 of the percent reduction from baseline are critical, with a lot of variation between countries. Could the authors provide some additional explanation on how these estimates were obtained.**

We agree. These calculations are described in lines 244-254 and now supplemented through equations in S4 File.

**Reviewer #5: This paper describes the total annual global cost of Plasmodium vivax malaria and estimates the cost savings associated with G6PD testing and radical cure assuming perfect and 30% adherence to treatment. This is an important topic and the total cost of Plasmodium vivax is under-studied. Though this could be considered as an additional cost-benefit model with little local implications, I fully agree with R1 that "producing such a paper based on macro-level evidence is challenging, and will always entail many trade-offs between availability and quality of data. I believe this is something we must accept and that research such as this has merit and is of potential importance for policy formulation. The value of such research, in my opinion, lies primarily in its ability to provide a birds perspective of important public health issues rather than representing exact information at local levels. With some methodological improvements". I thus also think that this paper is publishable.**

**The authors have also taken a careful attention to previous comments made by 3 knowledgeable reviewers and I think their answers are satisfactory. I liked the fact that they consider heterogeneity in their estimates (notably by age) and that they provide an online tool (with their assumptions).**

**My minor concerns related to this type of exercises are:**

**1/ The short-term horizon of the cost savings (one year) associated with the intervention or the full range of costs in the absence of intervention. Long-term versus short-term effects could be discussed more in the paper. In the absence of intervention, the long term effect may not be linear and could intensify or vanish over time.**

We agree that this is challenging and have added further details on this in the paragraph of study limitations (lines 483-488):

*“The scenario analyses only included costs over a one year time horizon; accordingly, relapses prevented beyond the time frame are not captured, thus underestimating the cost savings. The cost of scale up required to achieve provider compliance with G6PD screening and radical cure are also not included, underestimating the cost of implementation. Furthermore, the long-term effects are likely to fluctuate over time, particularly as countries near elimination and cases become rare events.”*

**2/ The range of costs considered in this study. Many economic studies have shown that vivax malaria control may have a broader range of economic consequences than the one analyzed in a cost-of-illness perspective. The authors do not make reference to such estimates and economic literature. The estimated costs thus certainly represent a low bound in this perspective. See e.g. and amongst other:**

**- Bleakley, H. (2010). `Malaria eradication in the Americas: A retrospective analysis of childhood exposure', American Economic Journal: Applied Economics, pp. 1{45.**

**- Burlando, A. (2015). `The Disease Environment, Schooling, and Development Outcomes: Evidence from Ethiopia', The Journal of Development Studies, vol. 51(12), pp. 1563{1584,ISSN 0022-0388, doi:10.1080/00220388.2015.1087512.**

**- Lucas, A.M. (2010). `Malaria eradication and educational attainment: evidence from Paraguay and Sri Lanka', American Economic Journal: Applied Economics, vol. 2(2), pp. 46{71.**

**- Lucas, A.M. (2013). `The impact of malaria eradication on fertility', Economic Development and Cultural Change, vol. 61(3), pp. 607{631.**

We agree that these are important consequences that are difficult to quantify and include in an analysis like this one. We now refer to this literature in lines 463-465:

*“It should be noted, however, that these calculations do not attempt to account for wider long-term economic impacts of disease, such as school performance [49,50], decreased fertility [51], and labor productivity [52].”*

**3/ The potential effects of interventions across areas with different institutions, different risks, different health systems (put differently, externalities or general equilibrium effects) that are only partially addressed.**

**These comments apply more generally to similar costs of illness studies, and model-based cost-benefits or cost-effectiveness analyses of this kind. It does not mean that they are not useful as mentioned above if one takes them with caution. I do think the discussion is sufficiently cautious here to avoid this caveat.**

We agree that it’s difficult to capture all of these issues in a global analysis and that countries will have to evaluate local scenarios within the context of their own health systems. We are glad that you find our discussion sufficiently nuanced on this point.
